# Spin-Based Quantum Energy Devices: From Quantum Thermal Machines to Quantum Batteries

**DOI:** 10.3390/e28040396

**Published:** 2026-04-01

**Authors:** Suman Chand, Riccardo Grazi, Niccolò Traverso Ziani, Dario Ferraro

**Affiliations:** 1 Center for Quantum Science and Technology, Siksha ‘O’ Anusandhan, Bhubaneswar 751030, Odisha, India; sumanchand@soa.ac.in; 2 Dipartimento di Fisica, Università di Genova, Via Dodecaneso 33, 16146 Genova, Italy; niccolo.traverso.ziani@unige.it (N.T.Z.); dario.ferraro@unige.it (D.F.); 3 CNR-SPIN, Via Dodecaneso 33, 16146 Genova, Italy

**Keywords:** quantum thermodynamics, quantum spin chains, quantum batteries

## Abstract

The progressive miniaturization of devices devoted to energy manipulation and storage calls for extending thermodynamic concepts towards regimes where quantum effects become unavoidable. In this context, quantum thermodynamics provides the proper framework for understanding and exploiting non-classical effects for energy applications. Within this framework, we present a comprehensive review of the role played by spin systems as versatile platforms for quantum energy technologies, focusing on their dual role as Quantum Thermal Machines and Quantum Batteries. We discuss how the combination of discrete spectra, engineered interactions and long coherence times enables the realization of high-performance quantum devices. We then highlight how genuinely quantum features can be exploited to achieve performance beyond classical limits. Beyond theoretical developments, we review the rapid experimental progress across leading spin platforms, including nuclear magnetic resonance systems, trapped ions, nitrogen-vacancy centers in diamond and superconducting circuits, which are bringing quantum energy devices from conceptual proposals to actual realizations. By presenting a unified spin-based framework that integrates energy conversion and storage, this review outlines the foundations of the emerging field of quantum energy and identifies key challenges and future directions for scalable quantum energy technologies.

## 1. Introduction

The need for miniaturized energy-processing devices has pushed modern technology towards a regime where quantum effects can no longer be neglected. Nanoscale engines, refrigerators, and energy storage devices are characterized by a limited number of active degrees of freedom, and their performance is crucially affected by fluctuations, coherences, and correlations [1]. This has motivated the emergence of the field of *quantum thermodynamics* as a theoretical and experimental framework that extends classical thermodynamic laws to out-of-equilibrium microscopic systems governed by quantum mechanics [2,3,4,5,6,7,8,9] and explores new routes toward quantum-enabled energy technologies [10].

At the quantum level, traditional concepts such as heat, work and entropy need to be properly reconsidered, as genuinely quantum resources—such as coherence and entanglement—become relevant for thermodynamical purposes. These features enable the realization of devices whose performance in terms of energy storage, efficiency and power can surpass classical limits under suitable operational constraints, while simultaneously raising fundamental questions about the ultimate bounds imposed by quantum mechanics [11,12,13,14,15]. As quantum effects become unavoidable, quantum thermodynamics naturally extends beyond the study of energy conversion alone to include the problem of storing and releasing energy in a controlled manner at the microscopic scale. This issue is driven by the requirements of emerging quantum devices, such as quantum computers, simulators and sensors, which demand coherent energy injection and extraction and therefore storage mechanisms that are intrinsically quantum [16].

In this broader context, spin systems have emerged as particularly versatile platforms for quantum thermodynamic applications [17]. Their discrete energy spectra and long coherence times, together with the high degree of experimental control achieved across a variety of experimental settings, make them ideal working media for implementing and experimentally testing quantum thermodynamic protocols across diverse physical platforms [18]. In this direction, great theoretical and experimental effort has been devoted in recent years to exploiting matter–radiation interaction for quantum thermodynamics purposes. Here, cavity quantum electrodynamics (QED) and circuit QED architectures have been proposed as a way to realize effective spin–spin interaction mediated by (real or virtual) photon exchange [19]. Such cavity-mediated spin systems have been widely investigated, in particular for quantum battery (QB) applications. Here, the cavity can act as a charger which transfers energy to a QB composed of independent two-level systems [20,21,22,23,24]. These devices are characterized by the emergence of collective quantum advantage in the charging power [25,26]. Newest developments in this domain have addressed, on the one hand, the optimization of the charging protocol via reinforcement learning [27,28,29] and on the other hand, an accurate investigation of the spectrum of the molecules playing the role of two-level systems to strongly enhance the energy storage time [30,31]. As stated above, apart from a few notable exceptions [29,32], these devices assume independent two-level systems. This hides many-body effects related to the direct spin–spin interaction. Conversely, interacting spin models provide direct access to collective effects and critical phenomena [33,34], while their controllable coupling to engineered reservoirs allows systematic investigation of dissipative and non-Markovian thermodynamic regimes [35,36]. These features enable the exploration of many-body quantum advantages relevant to different forms of quantum energy processing, including both conversion and storage [37,38,39,40,41,42,43,44].

Against this background, the central message of this work is that spin systems should be regarded not merely as a possible setting to implement specific thermodynamic cycles, but as a unified physical platform for quantum energy processing. While existing literature has often addressed quantum thermal machines (QTMs) and QBs as separate paradigms [18], here we show that spin systems naturally accommodate both functionalities within a single coherent framework. This unified viewpoint reveals deep connections between work extraction, coherence and collective many-body effects, providing a conceptual blueprint for the development of integrated quantum energy technologies.

The paper is organized as follows. In Section 2 we briefly review the fundamental laws of classical thermodynamics and their extension to the quantum regime. Section 3 discusses spin systems as quantum working media. Section 4 and Section 5 review spin-based QTMs and QBs, respectively. Section 6 presents experimental implementations, and Section 7 is devoted to conclusions.

## 2. From Classical to Quantum Thermodynamics

Before discussing spin-based quantum energy devices, we first review the basics of classical thermodynamics and its extension to the quantum regime. This provides the necessary background for understanding QTMs and QBs. We focus on heat, work, entropy, and extractable work (ergotropy), as these concepts are central to the rest of this review.

### 2.1. Laws of Classical Thermodynamics

Formalized more than two centuries ago, classical thermodynamics provides the theoretical foundation of modern technologies for energy manipulation, including heat engines, refrigerators, power plants and electrochemical batteries, to name just a few [45,46,47]. Originally elaborated for macroscopic systems, its laws define universal constraints on energy conversion, storage and dissipation. Reviewing these principles is therefore essential to establish the benchmarks against which quantum energy devices must ultimately be compared.

The so-called *zeroth law* introduces the notion of thermal equilibrium and defines the temperature (*T*) of a system. It also allows thermal reservoirs to be meaningfully characterized.

The *first law* expresses the conservation of energy and introduces the distinction between heat (*Q*) and work (*W*). It states that the infinitesimal change in the internal energy *U* of a system can be decomposed as

(1) dU=δQ−δW,

where δQ denotes the infinitesimal heat absorbed by the system and δW the infinitesimal work performed by the system on the surroundings. The notation emphasizes an important conceptual distinction: the internal energy *U* is a state function and therefore admits an exact differential dU, whereas heat and work are process-dependent quantities and are represented by inexact differentials δQ and δW. Their values depend on the specific thermodynamic path connecting two states rather than on the states themselves. For instance, in the absence of heat exchange (δQ=0), a positive δW reduces the internal energy, reflecting the fact that work output must be supplied by the system’s stored energy.

The *second law* introduces entropy (*S*), a state function that quantifies irreversibility. For a reversible process, its infinitesimal variation is given by

(2) $$dS=\frac{\delta Q_{\mathrm{rev}}}{T},$$

where $\delta Q_{\mathrm{rev}}$ denotes the infinitesimal heat exchanged along a reversible thermodynamic path at temperature *T*. For general (irreversible) processes, the entropy balance satisfies

(3) $$dS=\frac{\delta Q}{T}+dS_{\mathrm{prod}},\;dS_{\mathrm{prod}}\geq 0,$$

where $dS_{\mathrm{prod}}$ denotes the entropy production. For isolated systems (δQ=0), this implies dS≥0. Together with the first law, the second law sets fundamental limits on the performance of thermal machines, determines the direction of spontaneous processes and governs entropy production in physical and engineered systems.

The *third law*, also known as Nernst’s theorem, imposes fundamental limitations on cooling and refrigeration by stating that the absolute zero temperature cannot be reached in a finite number of steps. In other words, the cooling process requires increasingly more resources as the temperature decreases. This fundamental limitation restricts access to arbitrarily low cryogenic temperatures.

### 2.2. Classical Heat Engines and Refrigerators

Heat engines and refrigerators are the primary devices through which thermal energy is converted into useful work or controlled cooling. The simplest example of a heat engine operates between a hot reservoir at temperature $T_{H}$ and a cold reservoir at temperature $T_{C}$, absorbing heat $Q_{H}$ from the hot bath, performing work *W*, and releasing heat $Q_{C}$ to the cold bath. Its efficiency is defined as the ratio of work produced to the heat absorbed, namely

(4) $$\eta =\frac{W}{Q_{H}},$$

and is bounded by the *Carnot efficiency*,

(5) $$\eta_{\max}=1-\frac{T_{C}}{T_{H}}.$$

**Figure 1:**
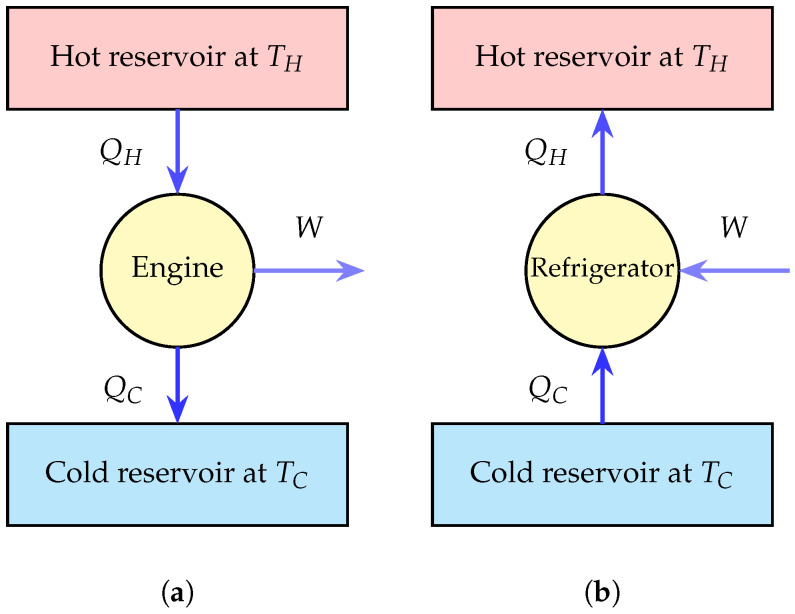
Schematic diagrams of (**a**) a heat engine and (**b**) a refrigerator. In a heat engine, heat $Q_{H}$ is absorbed from a hot reservoir at temperature $T_{H}$, part of it is converted into useful work *W* and the remaining heat $Q_{C}$ is released to a cold reservoir at temperature $T_{C}<T_{H}$. In a refrigerator, external work *W* is supplied to extract heat $Q_{C}$ from a cold reservoir and reject heat $Q_{H}=Q_{C}+W$ to a hot reservoir. These schematic representations form the basis for both classical and quantum thermal machines discussed in this review.

This bound is universal and independent of the working substance or engine design. A schematic representation of a heat engine and a refrigerator, highlighting heat and work flows between reservoirs, is shown in Figure 1. These classical thermodynamics considerations have inspired several canonical engine cycles including Carnot, Otto, Diesel, and Stirling, which form the backbone of modern power generation and conversion technologies [45,46,47].

Refrigerators and heat pumps operate in the reverse mode, using work to extract heat from cold reservoirs and maintain controlled low-temperature environments. Their performance is quantified by the coefficient of performance (COP) given by

(6) $$COP=\frac{|Q_{C}|}{W}\leq \frac{T_{C}}{T_{H}-T_{C}},$$

where the bound is again a consequence of the second law.

Reversible engines can achieve the maximum Carnot efficiency only in the quasistatic limit, namely when cycle durations become infinitely long and the output power (produced work over time) vanishes. However, real engines necessarily operate in finite time to deliver useful power, inevitably introducing irreversibility and entropy production. This leads to a fundamental trade-off between efficiency and power. A landmark result in this direction is the Curzon–Ahlborn efficiency [48]

(7) $$\tilde{\eta }=1-\sqrt{\frac{T_{C}}{T_{H}}}.$$

It represents a bound on the efficiency achievable at maximum power for a broad class of engines and provides realistic performance benchmarks for practical energy devices. The Curzon–Ahlborn efficiency arises in the so-called endoreversible regime [49], where internal transformations are assumed reversible while irreversibility is confined to finite-rate heat exchange with reservoirs. Finite-time thermodynamics [50] has since become a central framework for realistic energy devices, providing universal efficiency–power trade-offs relevant for technological applications.

Beyond energy transformation, classical thermodynamics is also characterized by a deep connection between information and work. This is epitomized by the idea of Maxwell’s demon and was later formalized through Landauer’s principle [51,52,53], which states that the erasure of one bit of information requires a minimum heat dissipation of $k_{B}T\ln2$ into a thermal reservoir at temperature *T*. This insight establishes information as a physical thermodynamic resource and anticipates the central role played by measurements and coherence in quantum thermodynamics. These classical bounds and trade-offs provide the reference framework within which QTMs must operate, although quantum coherence and correlations can modify the mechanisms through which such bounds are approached [4].

### 2.3. Why Quantum Energy Devices?

As energy devices are pushed toward the nanoscale, classical thermodynamics becomes insufficient to capture new effects inherent to microscopic systems such as fluctuations, coherence and correlations. These quantum features open new possibilities for enhanced energy conversion, storage and control. This motivates the study of QTMs and QBs, which aim to exploit genuinely quantum resources to surpass classical limitations while remaining consistent with thermodynamic laws.

Quantum thermodynamics extends its classical counterpart principles to out-of-equilibrium microscopic systems whose dynamics are governed by quantum mechanics. In this regime, energy exchange is influenced by quantum coherence and correlations. Moreover, in microscopic systems the relative magnitude of quantum and thermal fluctuations can become comparable to the mean values of thermodynamic observables, so that energy and entropy fluctuations are no longer negligible. As a result, thermodynamic quantities must be formulated, where possible, in terms of quantum states and operators. A quantum system is described by a generally time-dependent density operator ρ(t) and its internal energy at a given time is defined as the expectation value of its Hamiltonian H(t) according to

(8) U(t)=Tr[ρ(t)H(t)].

A finite variation of the internal energy ΔU over a given time range τ can be decomposed into heat and work contributions depending on whether they originate from state changes (heat) or Hamiltonian modulation (work), namely [54]

(9) $$\begin{array}{ccc} \Delta U & = & U(\tau )-U(0)\\ & = & \mathrm{Tr}[\rho (\tau )H(\tau )]-\mathrm{Tr}[\rho (0)H(0)]\\ & = & \int_{0}^{\tau }\mathrm{Tr}\left[\dot{\rho }\left(t\right)H\left(t\right)\right]dt+\int_{0}^{\tau }\mathrm{Tr}\left[\rho \left(t\right)\dot{H}\left(t\right)\right]dt\\ & \equiv & \langle Q\rangle +\langle W\rangle . \end{array}$$

In the quantum regime, heat and work remain process-dependent quantities, but are defined operationally through expectation values of the time-dependent state and Hamiltonian. This decomposition provides the operational basis to characterize quantum engines, refrigerators and charging protocols for quantum devices. Moreover, it highlights the process-dependent nature of the notions of heat and work also at the quantum level [55]. Here

(10) $$\langle W\rangle =\int_{0}^{\tau }\mathrm{Tr}\left[\rho \left(t\right)\;\dot{H}\left(t\right)\right]dt$$

represents the work performed on the system through external driving. With this convention, positive 〈W〉 increases the internal energy, in agreement with the classical first law dU=δQ−δW, where δW>0 denotes work performed by the system.

Concerning the entropy of a quantum system, it is quantified by the von Neumann formulation [56,57]

(11) $$S\left(\rho \right)=-k_{B}\mathrm{Tr}\left(\rho \ln\rho \right),$$

where $k_{B}$ is the Boltzmann constant, which reduces to the classical Gibbs entropy for equilibrium thermal states and characterizes the degree of mixedness of a quantum state.

According to the previous considerations, it is quite evident that the laws of thermodynamics remain valid also in quantum systems when properly reformulated. Indeed, the first law expresses conservation of energy using expectation values, while the second law constrains entropy production and defines efficiency bounds for quantum engines and refrigerators.

Beyond state functions, quantum thermodynamics is inherently operational and is formulated in terms of quantum thermodynamic processes, quantum analogues of classical thermodynamic transformations.

In a *quantum isothermal process*, the system remains in contact with a thermal reservoir at fixed temperature while its Hamiltonian is varied slowly. The system continuously relaxes to instantaneous thermal equilibrium, allowing simultaneous exchange of heat and work.

A *quantum adiabatic process* involves slow modulation of the Hamiltonian such that the system follows its instantaneous eigenstates. No heat is exchanged, and changes in energy arise solely from work. This follows directly from the definition of heat given in Equation (9). Indeed, during a quantum adiabatic process the system evolves unitarily under the time-dependent Hamiltonian, such that $\dot{\rho }\left(t\right)=-\frac{i}{\hbar }\left[H\left(t\right),\rho \left(t\right)\right]$. As a consequence, $\mathrm{Tr}[\dot{\rho }\left(t\right)H\left(t\right)]=0$ at all times, implying 〈Q〉=0. Therefore, any change in internal energy during an adiabatic stroke originates solely from work performed through the external modulation of the Hamiltonian. In finite-time operation, deviations from perfect adiabaticity generate coherence between instantaneous energy eigenstates, leading to internal friction and excess entropy production. At a microscopic level, this quantum internal friction originates from the non-commutativity of the driven Hamiltonian at different times, $[H\left(t\right),H\left(t^{\prime }\right)]\neq 0$, which induces non-adiabatic transitions during the work strokes [50,58,59,60].

In a *quantum isochoric process*, the Hamiltonian is kept fixed while the system interacts with a thermal bath. Energy exchange occurs purely as heat ($\dot{H}=0$ and consequently 〈W〉=0, see Equation (9)), leading to population redistribution and entropy change without work being performed.

Quantum *isobaric processes* maintain a constant generalized force, such as an external field gradient, while allowing both heat and work exchange. Although less commonly implemented, they complete the fundamental set of quantum thermodynamic transformations.

Together, these processes enable the construction of quantum thermodynamic cycles, such as Carnot, Otto, and Stirling cycles, using quantum working substances including spins, oscillators, and multilevel systems [50,61,62].

### 2.4. Thermodynamic Resources

We introduce here the operational quantities that will be used in the following to assess the energetic performance of quantum working media. A defining structural property of finite-dimensional systems (such as spin systems) is the boundedness of their energy spectrum. For a Hamiltonian *H* acting on a Hilbert space of dimension *d*, the spectrum consists of a finite set of eigenvalues ${\left\{E_{k}\right\}}_{k=1}^{d}$, implying the existence of a minimum and a maximum energy, $E_{\min}$ and $E_{\max}$. As a consequence, for any physical state ρ, one has

(12) $$E_{\min}\leq {\langle H\rangle }_{\rho }\leq E_{\max}.$$

This purely kinematic constraint sharply distinguishes finite-level systems from continuous-variable working media, such as harmonic oscillators or bosonic fields, whose spectra are unbounded from above. In a thermodynamic context, boundedness has immediate operational implications: it restricts not only the amount of energy that can be stored in the system, but also the maximum amount of work that can be extracted from it.

For isolated quantum systems, the relevant notion of work is therefore not the average energy itself, but the maximal energy that can be extracted through cyclic unitary transformations. These are operations where the Hamiltonian is modified only for a finite time before returning back to its initial value. This quantity is captured by the ergotropy [63,64,65]. Given a state ρ and Hamiltonian *H*, the *ergotropy* is defined as

(13) $$W\left(\rho ,H\right)=\mathrm{Tr}\left(\rho H\right)-\mathrm{Tr}\left(\pi_{\rho }H\right),$$

where $\pi_{\rho }$ denotes the passive state associated with ρ, obtained by rearranging the eigenvalues of ρ in decreasing order onto the energy eigenstates of *H* in increasing order [65]. *Passive states* are those from which no work can be extracted by any unitary operation acting on a single copy of the system. A stronger condition deriving from it is *complete passivity*: a state ρ is completely passive if no work can be extracted even from *n* independent copies, i.e., from $\rho^{⊗n}$, for any n≥1. It is possible to show that thermal Gibbs states, defined as

(14) $$\rho_{\beta }=\frac{e^{-\beta H}}{Z},$$

are completely passive for all inverse temperatures $\beta =1/k_{B}T$ [63,64]. In particular, complete passivity implies passivity, but the converse does not hold in general: a state may be passive (no work extractable from a single copy) yet not completely passive (work can be extracted from multiple copies via collective unitaries). As a direct consequence of the bounded spectrum, ergotropy obeys the absolute constraint

(15) $$W\left(\rho ,H\right)\leq E_{\max}-E_{\min},$$

which is purely kinematic and independent of the preparation protocol. This bound highlights the fact that increasing the average energy of a system does not necessarily increase the amount of extractable work; as for computing ergotropy, the detailed ordering of populations across the spectrum becomes fundamental. Energetic performance, therefore, cannot be assessed solely in terms of stored energy, but must explicitly account for passivity and ergotropy. Ergotropy therefore quantifies the maximum extractable work under unitary control, distinguishing usable energy from mere stored energy.

Another figure of merit which is relevant to characterize the functioning of QTMs and QBs is the *instantaneous power* given by [66,67]

(16) $$P\left(t\right)=\mathrm{Tr}\;\left[\rho \left(t\right)\;\frac{\partial H(t)}{\partial t}\right],$$

namely the time derivative of the total work defined in Equation (9).

The concepts introduced above provide the general thermodynamic framework within which quantum energy devices must be analyzed. In particular, the notions of bounded spectra, passivity, ergotropy, and controlled Hamiltonian modulation will play a central role in assessing the performance of spin-based quantum heat engines and QBs. We now specialize these general principles to spin systems, which constitute a versatile and experimentally accessible class of quantum working media.

## 3. Spin Systems as Working Media for Quantum Thermal Devices

Having established the general thermodynamic framework in terms of bounded spectra, ergotropy, and controlled Hamiltonian modulation, we now specialize these concepts to a concrete and experimentally relevant class of working media: interacting spin systems. Owing to their finite-dimensional Hilbert space, tunable interaction structure, and high degree of controllability, spin models provide a natural platform for implementing QTMs and QBs. In the following, we analyze how their spectral properties, symmetries, and openness influence thermodynamic performance.

### 3.1. Spin Hamiltonians and Controllable Energy Spectra

We consider *N* spin-1/2 degrees of freedom governed by the generic interacting Hamiltonian

(17) $$H_{S}=\sum_{i=1}^{N}\frac{\omega_{i}}{2}\sigma_{i}^{z}+\sum_{i<j}\sum_{\alpha =x,y,z}J_{ij}^{\alpha }\;\sigma_{i}^{\alpha }\sigma_{j}^{\alpha },$$

with $\sigma_{i}^{\alpha }$ the α-th Pauli matrix for a spin placed in the *i*-th lattice site, $\omega_{i}$ the level spacing of such two-level systems and $J_{ij}^{\alpha }$ a spin–spin coupling whose explicit form encodes the interaction range and characterize the specific model under investigation. The above general expression includes a wide class of experimentally relevant models such as quantum Ising, XXZ, Heisenberg and Kitaev-type Hamiltonians [33]. The structure of the many-body spectrum of spin Hamiltonians depends on both the pattern of interactions and the symmetries they respect. For instance, if the generic Hamiltonian in Equation (17) conserves the total magnetization

(18) $$S_{\mathrm{tot}}^{z}=\frac{1}{2}\sum_{i=1}^{N}\sigma_{i}^{z},\;\left[H_{S},S_{\mathrm{tot}}^{z}\right]=0,$$

where $S_{\mathrm{tot}}^{z}$ is the total magnetization operator along the *z* direction. If this symmetry is present, the Hilbert space can be decomposed into invariant magnetization sectors labeled by the eigenvalue *M* of $S_{\mathrm{tot}}^{z}$,

(19) $$H_{S}=\underset{M}{⨁}H_{S}^{\left(M\right)},$$

where ⨁ denotes a direct sum and $H_{S}^{\left(M\right)}$ acts within the subspace of fixed total magnetization *M*. Such symmetry-induced fragmentation constrains available transitions under physically realistic control operations, since coherent manipulations must either respect or explicitly break the corresponding conserved quantity.

Beyond symmetry, the structure of energy levels reflects whether a model is integrable or nonintegrable. Integrable spin chains [34], such as the isotropic Heisenberg (XXZ/XXX) family solvable by Bethe ansatz [68] or Jordan–Wigner transformations [69], exhibit an extensive set of conserved quantities and Poissonian level statistics [70]. Conversely, generic nonintegrable models display level repulsion and Wigner–Dyson statistics characteristic of chaotic many-body spectra [71,72,73]. This distinction can have deep consequences at the level of work extraction due to the fact that, while dense quasi-degenerate regions typical of integrable regimes permit finer population rearrangements at small energy increments, nonintegrable spectra with stronger level repulsion appear more rigid under unitary rearrangements.

Spatial locality and interaction range also shape spectral properties. For short-range interactions in one dimension, one has

(20) $$H_{S}=\sum_{i}h_{i}^{\left(1\right)}+\sum_{i}h_{i,i+1}^{\left(2\right)},$$

where $h_{i}^{\left(1\right)}$ are single-site (on-site) terms and $h_{i,i+1}^{\left(2\right)}$ describe nearest-neighbor interactions. In this case the total spectral bandwidth typically grows extensively,

(21) $$E_{\max}-E_{\min}=O\left(N\right),$$

where O(N) denotes linear scaling with system size *N*. In contrast, long-range or all-to-all couplings can modify the scaling of level spacings and bandwidth in nontrivial ways, potentially leading to super-extensive growth of stored energy or charging power at finite system size [37,74,75]. Recent analyses have further clarified that such anomalous scaling depends not only on interaction range but also on structural properties of the Hamiltonian, such as g-extensiveness, which quantifies the distribution of interaction energy across lattice sites [76].

Locality has further implications at the level of the control of the system’s state. Indeed, permutations of populations that are kinematically allowed may require long sequences of local operations when only nearest-neighbor interaction is exploitable, thereby constraining the operationally accessible ergotropy within finite time (finite number of operations) [77,78].

### 3.2. Open Spin Systems and System–Bath Interactions

Any realistic spin-based quantum thermal device is inherently open; namely, it interacts with uncontrolled degrees of freedom that act as an environment. From a microscopic perspective, this openness is encoded in a total (system+bath) Hamiltonian of the form

(22) $$H=H_{S}+H_{E}+H_{SE}$$

where $H_{S}$ is the spin Hamiltonian introduced above, $H_{E}$ describes the external environment (playing the role of thermal bath), and

(23) $$H_{SE}=\sum_{i,\alpha }S_{i}^{\alpha }⊗E_{i}^{\alpha }$$

represents the system–environment coupling, with $S_{i}^{\alpha }$ spin operators defined in the Hilbert space of the system and $E_{i}^{\alpha }$ generic operators in the Hilbert space of the environment [79,80]. The central theoretical question is how the microscopic interaction between system and environment translates into an effective dynamical description for the reduced state $\rho_{S}\left(t\right)={\mathrm{Tr}}_{E}\left[\rho_{SE}\left(t\right)\right]$ of the spin working medium, where $\rho_{SE}\left(t\right)$ is the density matrix of the complete system.

The most widely used framework to approach this kind of problem in quantum thermodynamics is the *Markovian approximation*, which assumes weak system–environment coupling, fast decay of environmental correlations and a clear separation between system and environment timescales. Under these conditions, the reduced dynamics of the spin system is governed by a time-homogeneous master equation of the Lindblad form [79,81,82],

(24) $$\frac{d\rho_{S}}{dt}=-i\left[H_{S}+H_{\mathrm{LS}},\rho_{S}\right]+D\left[\rho_{S}\right],$$

where $H_{\mathrm{LS}}$ is the Lamb-shift Hamiltonian and D is a completely positive, trace-preserving dissipator. This latter accounts for irreversible processes induced by the environment and can be written in the standard form (for more details see [79])

(25) $$D\left[\rho_{S}\right]=\sum_{\omega ,\alpha }\gamma_{\alpha }\left(\omega \right)\left(A_{\alpha }\left(\omega \right)\rho_{S}A_{\alpha }^{†}\left(\omega \right)-\frac{1}{2}\left\{A_{\alpha }^{†}\left(\omega \right)A_{\alpha }\left(\omega \right),\rho_{S}\right\}\right),$$

where $A_{\alpha }\left(\omega \right)$ are jump operators associated with transitions between energy eigenstates of $H_{S}$ in its energy eigenbasis, separated by Bohr frequencies ω, and $\gamma_{\alpha }\left(\omega \right)$ are bath-induced transition rates determined by the spectral density and temperature of the environment [66,79].

A defining property of Markovian dynamics is the absence of memory effects. Within this picture, the evolution of $\rho_{S}\left(t\right)$ depends only on its instantaneous state, not on the previous history. This property guarantees thermodynamic consistency under standard assumptions, including monotonic entropy production and well-defined heat and work fluxes, provided that the bath remains close to thermal equilibrium [50,66,79,83].

In the Markovian regime, the interaction with a thermal bath typically drives the spin system toward a stationary state that satisfies detailed balance with respect to $H_{S}$,

(26) $$\frac{\gamma_{\alpha }\left(\omega \right)}{\gamma_{\alpha }\left(-\omega \right)}=e^{\beta \omega },$$

where $\beta =1/(k_{B}T)$ is the inverse temperature of the thermal bath at temperature *T*. As a consequence, the steady state is often a Gibbs state or, more generally, block-diagonal in the energy eigenbasis, with memoryless relaxation naturally leading them to passive states (from which no work can be extracted by a single-copy unitary) or completely passive states (from which no work can be extracted even from an arbitrarily many-copy unitary) [63,64].

However, many experimentally relevant spin platforms operate outside the strict Markovian regime. Strong system–bath coupling, structured reservoirs, finite-size environments or slow evolution of the bath correlations invalidates the assumptions underlying Equation (24), leading to so-called *non-Markovian dynamics*. In this case, the decay (transition) rates $\gamma_{\alpha }\left(\omega ,t\right)$ appearing in the dissipator become time-dependent and may temporarily take negative values. Such negative rates indicate a backflow of information from the environment to the system, corresponding to a temporary “recover” of coherence or population. Formally, this signals a breakdown of complete positive (CP) divisibility of the evolution [84], meaning that it can no longer be divided into infinitesimal, independent steps without losing the positivity of the state. This time-local formulation remains valid provided that system–bath correlations decay on timescales that are slow compared to the intrinsic system dynamics, allowing for an adiabatic elimination of bath degrees of freedom. In regimes of very strong coupling or long-lived correlations, more general non-Markovian approaches such as Nakajima–Zwanzig integro-differential equations, hierarchical equations of motion or Caldeira–Leggett approach are needed [79,85,86,87,88].

From a thermodynamic standpoint, non-Markovianity profoundly alters relaxation and energy-exchange processes. Memory effects can slow down thermalization, generate transient coherences in the energy eigenbasis or even induce revivals of ergotropy that are impossible in strictly Markovian settings [89]. As a result, the conventional intuition that dissipation monotonically degrades thermodynamic performance no longer holds.

For QTMs, non-Markovian dynamics can enhance power output by temporarily storing energy in system–environment correlations and releasing it back into the working medium during the cycle [90]. Similarly, in QBs, memory effects can stabilize non-passive states against relaxation, effectively prolonging storage times and increasing the accessible ergotropy [91].

In the Lindblad description, dissipation is encoded through jump operators $A_{\alpha }$, which represent elementary system–environment exchange processes such as energy relaxation, excitation, or dephasing acting on the spin degrees of freedom. Importantly, whether dissipation appears as local or collective at the level of these jump operators is secondary to the presence or absence of memory: both local and collective couplings can give rise to Markovian or non-Markovian dynamics, depending on the bath spectral density and correlation times [36,79,87]. From a thermodynamic perspective, it is therefore the Markovian versus non-Markovian character of the reduced dynamics that constitutes the primary organizing principle.

### 3.3. Coherence and Correlations in Spin Working Media

*Quantum coherence* and *correlations* are typically generated during the evolution of spin-based thermal devices. In fact, time-dependent driving and many-body interactions, as well as dissipation if the system is open, typically produce states that are not diagonal in the energy eigenbasis, even if the initial state is thermal or more generally passive. To assess the thermodynamic relevance of such features, it is important to distinguish between coherence in the energy eigenbasis and within degenerate subspaces. Writing the system Hamiltonian as

(27) $$H_{S}=\sum_{k}E_{k}|E_{k}\rangle \langle E_{k}|,$$

any density matrix describing the state can be decomposed as

(28) $$\rho_{S}=\sum_{k}\rho_{kk}|E_{k}\rangle \langle E_{k}|+\sum_{k\neq l}\rho_{kl}|E_{k}\rangle \langle E_{l}|.$$

In this case, the off-diagonal terms include both coherences between different energy eigenspaces and coherences within the same degenerate subspace. Only coherence between non-degenerate energy eigenstates can modify the ergotropy. Coherence within degenerate eigenspaces is thermodynamically inert and cannot be converted into work [12,92]. More generally, since we stated that ergotropy depends solely on the eigenvalues of $\rho_{S}$ and their ordering relative to the energy spectrum, coherence alone does not guarantee any thermodynamic advantage and may become irrelevant once control costs are properly accounted for [93]. Here, control costs refer to the physical resources required to implement the unitary operations needed to exploit coherent degrees of freedom, such as time-dependent driving, precision, and external work sources.

Correlations introduce an additional layer of complexity. To clarify the role of interactions, consider two subsystems with local Hamiltonians $H_{1}$ and $H_{2}$. If the total Hamiltonian is additive,

(29) $$H_{S}=H_{1}+H_{2},$$

meaning that the subsystems do not interact, and if the global state is a product state $\rho_{12}=\rho_{1}⊗\rho_{2}$, then ergotropy is additive and satisfies

(30) $$W\left(\rho_{1}⊗\rho_{2},H_{1}+H_{2}\right)=W\left(\rho_{1},H_{1}\right)+W\left(\rho_{2},H_{2}\right).$$

Thus, in this noninteracting and uncorrelated case, no additional extractable work arises from combining the subsystems.

However, if the composite state $\rho_{12}$ contains classical or quantum correlations, or if the Hamiltonian includes interaction terms beyond $H_{1}+H_{2}$, this additivity generally breaks down. This leads to a correlation-induced contribution to the ergotropy

(31) $$\Delta W_{\mathrm{corr}}=W\left(\rho_{12},H_{S}\right)-W\left(\rho_{1},H_{1}\right)-W\left(\rho_{2},H_{2}\right),$$

which can be either positive or negative. Here $\rho_{12}$ denotes the density matrix of the composite system, while $\rho_{1}={\mathrm{Tr}}_{2}\left[\rho_{12}\right]$ and $\rho_{2}={\mathrm{Tr}}_{1}\left[\rho_{12}\right]$ are the reduced states of subsystems 1 and 2, governed by Hamiltonians $H_{1}$ and $H_{2}$, respectively. One finds that correlations can either enhance or suppress extractable work, depending on how populations are distributed across the many-body energy spectrum [12]. This observation motivates a fundamental distinction between kinematic and dynamical advantages in energy extraction. Kinematic advantages arise from the enlarged set of accessible unitaries when global operations on the composite system are allowed, while dynamical advantages originate from interaction-driven processes that generate states inaccessible to parallel single-spin protocols [3,37,94].

The structural features discussed above, such as spectral controllability, locality, openness, coherence, and correlations, determine how energy is stored, transferred, and extracted in spin systems. These properties directly influence both the efficiency and the power of quantum thermal devices. We now proceed to the explicit construction of spin-based QTMs, where these general principles are implemented in cyclic and continuous engine architectures.

## 4. Spin-Based Quantum Thermal Machines: The Quantum Otto Engine Case

Having outlined the thermodynamic properties of spin systems, including bounded spectra, symmetry-induced sectorization, openness, and the role of coherence and correlations, we now examine their operation as QTMs. Spin systems provide a versatile platform where Hamiltonian modulation, engineered dissipation, and many-body interactions can be combined to implement controlled energy conversion cycles. In this section, we review cyclic, autonomous, and measurement-based realizations of spin quantum heat engines, highlighting how quantum resources modify performance and operational principles.

### 4.1. Cyclic Engines

Spin-based QTMs realized using finite-dimensional spin systems as working media can be externally modulated by acting on tunable control parameters such as a magnetic field or interaction strength. When the spin system operates as a QTMs, it plays the role of a *working medium*. To emphasize this operational function, we denote its Hamiltonian by $H_{\mathrm{WM}}\left(\xi \right)$, where ξ denotes an externally controllable spectral parameter, such as a magnetic field amplitude or interaction strength, which modulates the energy gaps of the working medium. In the instantaneous energy eigenbasis, it can be written as

(32) $$H_{\mathrm{WM}}\left(\xi \right)=\sum_{n}E_{n}\left(\xi \right)|n(\xi )\rangle \langle n(\xi )|.$$

During thermodynamic cycles, the periodic modulation of ξ induces work exchange, while thermalization is responsible for heat flow between the working medium and reservoirs used as thermal bath [9,54,95]. This structure establishes a formal correspondence with QB charging protocols, where energy injection similarly proceeds via controlled modifications of the system Hamiltonian. As a result, spin QTMs and QBs share a common energetic backbone, enabling a unified thermodynamic framework for quantum energy conversion and storage.

**Figure 2:**
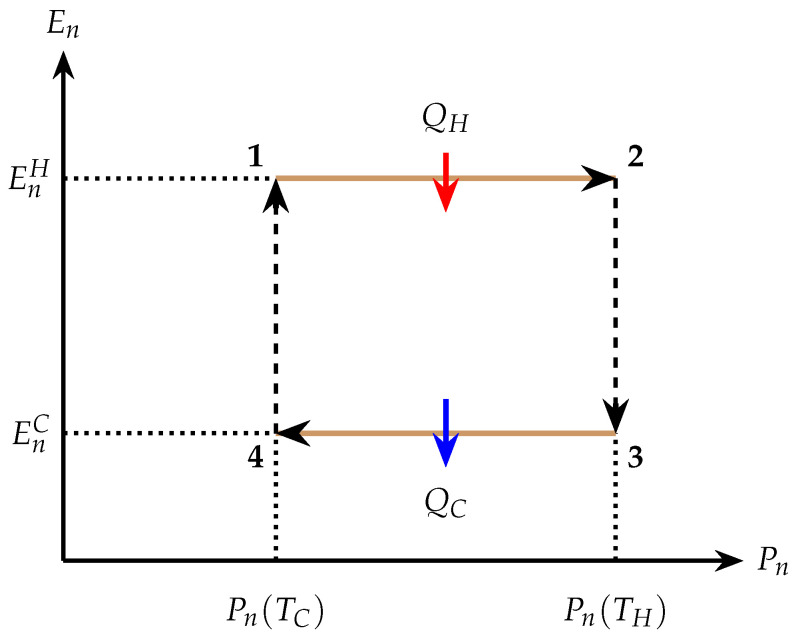
Schematic energy–population ($E_{n}$–$P_{n}$) representation of a quantum Otto cycle for a spin-based working medium governed by $H_{\mathrm{WM}}\left(\xi \right)$. The vertical axis shows the instantaneous energy levels $E_{n}\left(\xi \right)$ controlled by an external parameter ξ, corresponding to the hot ($\xi_{H}$) and cold ($\xi_{C}$) configurations shown as $E_{n}^{H}$ and $E_{n}^{C}$ in the diagram. The horizontal axis denotes the corresponding occupation probabilities $P_{n}$. Isochoric strokes (solid horizontal lines) occur at fixed spectra and involve heat exchange with a hot ($Q_{H}$) or cold ($Q_{C}$) reservoir through population redistribution, where $P_{n}\left(T_{H}\right)$ and $P_{n}\left(T_{C}\right)$ denote thermal populations at temperatures $T_{H}$ and $T_{C}$ ($T_{C}<T_{H}$), respectively. Adiabatic strokes (dashed vertical lines) deform the energy spectrum unitarily at fixed populations, resulting in work exchange. The four points represent the states of the cycle: (1) initial state before contact with the hot reservoir, (2) after isochoric heating and thermalization at temperature $T_{H}$, (3) after adiabatic expansion, and (4) after isochoric cooling at temperature $T_{C}$. The cycle is completed by an adiabatic compression stroke that brings the system back to the initial state (1). The cycle clearly illustrates the separation of heat and work characteristic of quantum Otto engines.

In contrast to Carnot cycles, where heat and work are exchanged simultaneously during isothermal transformations, the *quantum Otto cycle* [61,62,96,97,98,99,100,101] is a four-stroke engine in which heat and work exchanges occur in distinct strokes, i.e., well-defined thermodynamic transformations forming the cycle. The quantum Otto cycle is a four-stroke engine composed of: (i) an isochoric heating stroke, where the spin system is coupled to a hot bath and populations thermalize at a fixed energy spectrum; (ii) an adiabatic expansion stroke, where the Hamiltonian parameter ξ is varied unitarily, changing the energy gaps without heat exchange; (iii) an isochoric cooling stroke, where the system releases heat to a cold bath at a fixed spectrum; and (iv) an adiabatic compression stroke that restores the original Hamiltonian. A schematic representation of the quantum Otto cycle in the energy–population ($E_{n}-P_{n}$) plane is shown in Figure 2. This diagram provides an intuitive visualization of how heat exchange arises from population changes at fixed spectra during isochoric strokes, while work extraction originates from adiabatic deformations of the energy levels at constant populations. Such a separation of energetic contributions is particularly transparent in finite-dimensional spin systems and forms the basis for analyzing both single-particle and many-body quantum heat engines. In this representation, heat exchanged with the reservoirs is associated with changes in occupation probabilities at fixed energies,

(33) $$Q=\sum_{n}E_{n}\Delta P_{n},$$

while work performed during adiabatic strokes arises from spectral deformations at fixed populations,

(34) $$W=\sum_{n}P_{n}\Delta E_{n}.$$

These expressions follow from the quantum first law $\Delta U=\sum_{n}E_{n}\Delta P_{n}+\sum_{n}P_{n}\Delta E_{n}$, where during isochoric strokes $\Delta E_{n}=0$ and during adiabatic strokes $\Delta P_{n}=0$. These expressions assume weak system–bath coupling and negligible interaction energy. Depending on the direction of heat and work flows, the same four-stroke cycle operates either as a heat engine or as a refrigerator [95,102,103].

Interacting spin ensembles constitute genuine many-body working media whose thermodynamic behavior is qualitatively distinct from that of independent spins. Indeed, interactions reshape the energy spectrum, generate entanglement during work strokes and induce cooperative heat transport, potentially enhancing output power and modifying efficiency–power trade-offs. In this direction, collective spin engines based on Heisenberg [97,98,99,101,104,105,106,107], XXZ [108,109,110], XX [104], XY [111,112,113], and Lipkin–Meshkov–Glick [114,115,116] Hamiltonians have demonstrated regimes of superlinear scaling of output power under global driving protocols [20,117,118]. The mechanisms responsible for enhanced performance in many-body QTMs have direct conceptual analogues in QBs. In QTMs, cooperative transitions and interaction-induced correlations can enhance heat currents and work extraction rates beyond the independent-particle limit. In QBs, similar collective dynamics can instead accelerate the charging process, leading to superlinear scaling of the charging power with the number of battery cells [20,37]. In both QTMs and QBs, the underlying physical resource is the same: the ability of many-body interactions to generate collective excitations and correlated transitions that redistribute energy efficiently across the system. This correspondence highlights a deeper conceptual unity between QTMs and QBs, where the same many-body mechanisms can be exploited either for energy conversion or for energy storage, depending on the operational protocol. In these systems, cooperative transitions allow heat currents and work extraction rates to scale faster than linearly with the number of spins, providing a thermodynamic analogue of the collective charging advantages observed in QBs (see below). Such collective spin QTMs can be described by general interacting Hamiltonians of the form introduced in Equation (17), where interaction-induced collective excitations enable simultaneous multi-spin transitions and enhanced work extraction. These effects highlight entanglement and many-body correlations as genuine thermodynamic resources.

Quantum criticality due to many-body interactions can also enhance thermodynamic performance in spin heat engines. Indeed, when operating near quantum phase transitions, spin systems exhibit diverging susceptibilities and vanishing energy gaps, leading to amplified heat currents, enhanced work fluctuations and modified efficiency bounds [117,119,120]. Engines operating close to critical points can exploit these singular responses to achieve enhanced power output and robustness against parameter fluctuations.

On a different front, strong-coupling [121,122,123,124,125] and memory effects associated with the environment can introduce qualitatively new thermodynamic regimes. In fact, unlike Markovian reservoirs, non-Markovian baths exhibit memory effects and information backflow that can temporarily enhance heat currents and allow transient violations of standard power bounds [90,125,126,127,128,129,130,131]. Also, in this case, spin systems provide an experimentally viable platform to explore such regimes due to their tunable couplings and engineered reservoirs.

### 4.2. Autonomous Heat Engines

Beyond externally driven cyclic machines, a rapidly growing class of new QTMs has recently emerged, where the entire thermodynamic cycle is realized by means of fixed system–bath couplings without the need of time-dependent classical control [102,132,133,134,135]. In these machines, usually dubbed *autonomous heat engines*, the working medium is permanently coupled to multiple reservoirs, and work is stored into auxiliary quantum degrees of freedom (often another spin, a harmonic oscillator or cavity mode), forming a self-contained quantum engine. Cyclic quantum heat engines correspond to reciprocating machines in classical thermodynamics, whereas autonomous engines represent their continuous (non-reciprocating) counterparts.

A generic autonomous spin engine is described by a generalization of Equation (22) of the form

(35) $$H=H_{\mathrm{WM}}+\sum_{l}\left(H_{E_{l}}+H_{SE_{l}}\right)+H_{W}$$

where $H_{W}$ may represent a harmonic oscillator, cavity mode, or auxiliary spin acting as a quantum work repository that stores extracted energy, and the index *l* runs over the various environments coupled to the system. Heat currents from the baths drive population inversion or coherence generation inside the spin working medium, which is coherently transferred to the work repository without any external driving.

Such devices operate as continuous QTMs, delivering steady-state power rather than cyclic output [50,135,136]. Importantly, the extracted work can be stored directly into a spin-chain battery, creating a fully autonomous quantum engine–battery unit [137]. Such hybrid architectures naturally connect energy conversion and energy storage in the quantum regime. In these integrated systems, the output of the autonomous heat engine can be stored directly as ergotropy in a QB, eliminating the need for an intermediate classical work repository [137,138,139]. This unified framework highlights how quantum thermodynamic devices can operate as complete energy-processing units, where heat-to-work conversion and work storage occur within the same coherent quantum architecture. In this architecture, thermal gradients are converted into coherent many-body excitations stored as ergotropy inside a QB, without any classical clock, feedback loop or measurement intervention. Such machines realize fully quantum-coherent power generation mechanisms and constitute the quantum analogue of autonomous power plants.

### 4.3. Measurement-Based Quantum Heat Engines

A conceptually distinct class of QTMs is that of *measurement-based quantum heat engines*. Here, quantum measurements act as active thermodynamic resources rather than passive probes. In these devices, energy exchange is driven by the non-unitary back-action associated with quantum measurements, which can supplement or partially replace conventional thermal reservoirs. The thermodynamic role of measurement and feedback was established in [140,141,142].

Early implementations of measurement-based quantum heat engines were developed in the context of spin and trapped-ion working media, where it was shown that projective measurements can induce controlled energy changes and enable heat-engine operation without requiring a conventional cold bath [103,143]. In these implementations, the working medium was continuously or intermittently coupled to a thermal reservoir, while measurements performed on the system or on ancillary degrees of freedom effectively play the role of a second reservoir by injecting or extracting energy through measurement back-action. Related measurement-powered and feedback-assisted quantum engines have been investigated in Refs. [110,144,145,146,147,148,149,150,151,152,153,154], highlighting measurement back-action as a thermodynamic resource.

From a thermodynamic perspective, measurements can generate non-passive quantum states with finite ergotropy, which can subsequently be converted into useful work during unitary strokes. This places measurement-based engines naturally within the ergotropy framework adopted in this review and highlights a fundamental distinction between stored energy and extractable work. Importantly, the measurement-induced energy exchange is accompanied by entropy production associated with information gain and apparatus resetting, ensuring consistency with the laws of thermodynamics [146,155].

Subsequent developments have extended the measurement-based framework to interacting spin systems and finite-time operation. In particular, it has been shown that continuous system–bath coupling, when combined with appropriately timed measurements, leads to rich non-equilibrium behavior, including oscillatory efficiency, coherence-assisted work extraction and enhanced performance near quantum critical points [120]. These effects arise from the interplay between measurement back-action, many-body interactions and finite-time dynamics.

Measurement-based engines differ conceptually from autonomous quantum heat engines. While autonomous machines rely on steady-state energy currents between multiple reservoirs, measurement-based devices exploit the intrinsically quantum, non-unitary nature of measurement as a controllable thermodynamic resource. Their operation is intrinsically discrete and intervention-based: thermodynamic transformations are triggered by measurement events rather than sustained by continuous reservoir-induced currents.

Overall, measurement-based quantum heat engines establish a direct link between quantum measurement theory, information thermodynamics and energy conversion. Spin systems, with their tunable interactions and well-controlled measurement protocols, provide a versatile platform to explore this paradigm and its implications.

The different paradigms discussed above, namely cyclic, autonomous, and measurement-based quantum heat engines, show that spin systems can transform thermal or informational resources into usable work through controlled Hamiltonian modulation, engineered dissipation, and quantum measurements. In all these architectures, work extraction ultimately corresponds to the controlled manipulation of populations and coherences within a bounded energy spectrum. This observation naturally leads to a related question: how can the injected or extracted energy be stored and later recovered in a controlled and efficient way? We now address this issue by examining spin systems functioning as QBs, where the emphasis shifts from energy conversion to energy storage and the optimization of ergotropy.

## 5. Spin-Based Quantum Batteries

In the previous sections, we introduced the operational framework of quantum thermodynamics and discussed how quantum systems can be exploited to convert energy into different forms, with particular emphasis on spin-based QTMs. Here, we turn from energy conversion to the complementary task of quantum energy storage and controlled release. To address this topic, the notion of QBs was first formalized in 2013 by Alicki and Fannes [156], who proposed finite-dimensional quantum systems as microscopic energy-storage devices. In contrast to classical batteries, such as electrochemical cells and capacitors, which store energy in non-equilibrium chemical or electrical states, QBs store energy in quantum states of microscopic systems and release it through controlled unitary or dissipative dynamics. Since their introduction, QBs have attracted growing attention for different reasons: first, these devices can be studied as a testing ground for genuinely quantum effects in thermodynamics like coherence, correlations and many-body phenomena. Second, as we will discuss in this section, quantum properties can allow QBs to outperform their classical counterparts, for example, in terms of charging power. Finally, QBs are not simply nanoscale versions of classical batteries, but they represent a genuinely new paradigm for energy storage. In this sense, even the simplest building block of quantum technologies, a single qubit, can in principle be viewed as a QB. From this perspective, the ground (excited) state of a qubit can be associated with an empty (fully charged) battery. The transition from the ground to the excited state naturally plays the role of the charging process. Formally, a spin-based QB differs from a spin-based QTM only in its operational objective: while QTMs convert heat into work through cyclic dynamics, QBs aim to store work in non-passive states characterized by finite ergotropy.

**Figure 3:**
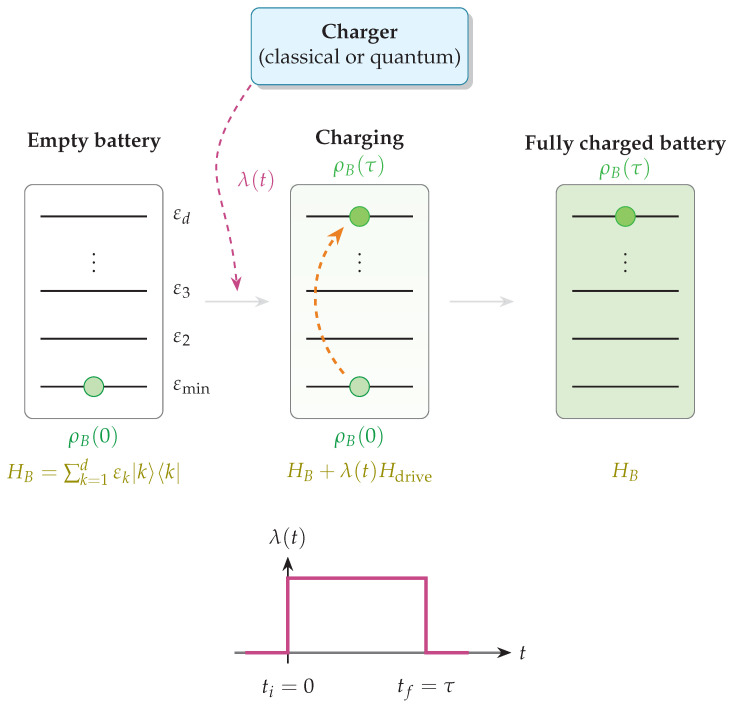
Schematic illustration of a QB charging protocol in a spin-based system. Left: an initially empty battery described by a nondegenerate Hamiltonian $H_{B}=\sum_{k=1}^{d}\varepsilon_{k}|k\rangle \langle k|$, where $\varepsilon_{\min}$ and $\varepsilon_{d}$ denote the ground-state and highest energy levels, respectively. The battery is prepared in the ground state $\rho_{B}\left(0\right)$ (light green). Middle: during the charging stage, the battery is coupled to a classical or quantum charger through an interaction driven by the time-dependent coupling λ(t), leading to an effective Hamiltonian $H_{B}+\lambda \left(t\right)H_{\mathrm{drive}}$ in the classical scenario and inducing population transfer across the energy levels. Right: after disconnecting the charger, the battery returns to its bare Hamiltonian $H_{B}$ and reaches a charged state $\rho_{B}\left(\tau \right)$ (dark green) satisfying $\mathrm{Tr}\left[H_{B}\rho_{B}\left(\tau \right)\right]>\varepsilon_{\min}$.

Since two-level systems constitute the simplest realization of QBs [157,158,159], spin chains have emerged as a paradigmatic platform for their study, as they provide a direct many-body extension in which interactions, correlations and energy transport can be systematically explored, while remaining directly relevant to experimental platforms ranging from nuclear magnetic resonance [160,161] to solid-state and superconducting qubits [162,163]. When the same spin architecture is used as a QB, we denote its Hamiltonian by $H_{B}$ to emphasize its storage function. The internal Hamiltonian of a QB can be written in general as

(36) $$H_{B}=\sum_{k=1}^{d}\varepsilon_{k}|k\rangle \langle k|$$

where {|k〉} denotes the energy eigenstates of the battery Hamiltonian, and the energy levels are assumed to be nondegenerate for the sake of simplicity. *Charging* the QB prepares the system in a certain excited state $\rho_{B}\left(\tau \right)$ such that $\mathrm{Tr}\left[H_{B}\rho_{B}\left(\tau \right)\right]>\varepsilon_{\min}$. A representative charging protocol is illustrated schematically in Figure 3, highlighting the transition from an unchanged battery to a charged state through a finite-time interaction with a charger; here, a time-dependent parameter λ(t) is switched on at time $t_{i}=0$ and then switched off at time $t_{f}=\tau$ [16,164]. During this time interval, the quantum system that takes the role of the battery interacts with a classical or quantum system that acts as a charger, enabling the charging of the device.

### 5.1. Thermodynamic Resources for Quantum Batteries

In this section, we discuss the behavior of the energy stored, ergotropy and power (see Equations (9), (13) and (16)) characterizing the specific case of quantum spin systems employed as QBs. To start, we consider here, for the sake of simplicity, the case of a classical charger (formulations with quantum chargers are also possible), following the protocol described in the previous subsection and reported in Figure 3. Suppose the QB is initially prepared in a state $\rho_{B}\left(t=0\right)=\rho_{B}\left(0\right)$. Its evolution during any unitary charging or discharging process can then be described directly in terms of its own density matrix under an effective Hamiltonian

(37) $$\dot{\rho_{B}}\left(t\right)=-i\left[H_{B}+\lambda \left(t\right)H_{\mathrm{drive}},\;\rho_{B}\left(t\right)\right],$$

where *ħ* has been set to unity and $\lambda \left(t\right)H_{\mathrm{drive}}$ represents here the action of a classical charger whose dynamics is not affected by that of the QB. In contrast, for a fully quantum charger, one would consider the combined battery–charger system with Hamiltonian $H_{B}+H_{C}+\lambda \left(t\right)H_{BC}$, where $H_{C}$ denotes the charger Hamiltonian and $H_{BC}$ the battery–charger interaction. The battery state would then be obtained as the partial trace over the charger degrees of freedom, $\rho_{B}\left(t\right)={\mathrm{Tr}}_{C}\left[\rho_{BC}\left(t\right)\right]$. At time t=τ, the battery gets disconnected from the charger and its state is described by the density matrix $\rho_{B}\left(\tau \right)$. The *energy stored* in the QB after the charging process, generically given by Equation (9), becomes

(38) $$\Delta E\left(\tau \right)=\mathrm{Tr}\left[\left(\rho_{B}\left(\tau \right)-\rho_{B}\left(0\right)\right)H_{B}\right].$$

Such quantity and associated ergotropy (see Equation (13)) represent the principal figures of merit for the unitary work injection and extraction capacity of the device. From this perspective, many-body batteries can exhibit an advantage over single-body ones: it is possible to extract work from the ensemble even if each individual subsystem is in a passive state, provided that the overall state is not completely passive.

It is important to stress the conceptual difference between *classical* and *quantum chargers*. In the classical case, the charging term $\lambda \left(t\right)H_{\mathrm{drive}}$ represents an externally controlled time-dependent drive acting directly on the battery Hilbert space. The charger is not treated as a dynamical quantum system and no entanglement or back-action occurs. In this case, the battery evolution is described by a closed, unitary dynamics generated by an externally prescribed time-dependent Hamiltonian. In contrast, for a fully quantum charger, $H_{C}$ describes an independent quantum system and $H_{BC}$ the battery–charger interaction. The global evolution is unitary in the joint battery–charger Hilbert space, but the reduced battery state $\rho_{B}\left(t\right)$ generally becomes mixed due to entanglement generated with the charger, and its dynamics is described by a completely positive trace-preserving map rather than a unitary evolution. As a consequence, classical and quantum charging schemes may lead to quantitatively and qualitatively different charging dynamics, power scaling, and ergotropy accumulation.

During the dynamical evolution, the instantaneous charging power of the battery can be monitored, as defined in Equation (16). Integrating P(t) over the duration of the protocol gives the *average charging power*

(39) $$\bar{P}=\frac{\Delta E(\tau )}{\tau },$$

while an analogous quantity can be defined in terms of the ergotropy,

(40) $${\bar{P}}_{W}=\frac{W(\rho_{B}\left(\tau \right),H_{B})}{\tau },$$

which quantifies the average rate at which extractable work is accumulated in the battery. From an experimental perspective, the aim is to maximize this quantity, which naturally translates into the design of charging protocols that operate as fast as possible. However, note that the unitary operations that have been described to charge the QB cannot be performed instantaneously nor at arbitrarily short times because of the unavoidable constraints imposed by the so-called *quantum speed limit* (QSL) [165], which derives from the time–energy uncertainty relations and determines the minimal time required to reach some target state $\rho_{B}\left(\tau \right)$ starting from some initial state $\rho_{B}\left(0\right)$ by means of unitary evolution. If both initial $|\psi_{i}\rangle$ and final states $|\psi_{f}\rangle$ are pure, the QSL can be expressed in terms of the Mandelstam–Tamm and Margolus–Levitin bounds [165], leading to

(41) $$\tau_{\mathrm{QSL}}=\frac{\;\arccos\;\left(|\langle \psi_{i}|\psi_{f}\rangle |\right)}{\min\;\left\{\langle \Delta H_{B}\rangle ,\langle H_{B}\rangle -E_{0}\right\}},$$

where $E_{0}$ denotes the ground-state energy of $H_{B}$. Here the bound is expressed in terms of the effective Hamiltonian governing the battery dynamics. For simplicity, we write it in terms of $H_{B}$, assuming bounded driving strength. In this context, optimal control theory provides a systematic framework to design charging protocols that approach or even saturate the QSL bound. Rather than relying on fixed, time-independent driving, optimal control methods allow one to tailor the time profile of the Hamiltonian parameters, such as the driving field amplitude and phase, so as to minimize the charging time while satisfying physical constraints. This approach has been shown to yield significant improvements in charging speed for both single- and multi-qubit battery models [166,167].

If the QB is a closed system and only unitary protocols are described, the amount of work *W* extracted is equal in magnitude to the stored energy ΔE. However, for non-unitary protocols or open batteries (that we will discuss later), it becomes important to quantify how much work can be extracted from such a QB. As we discussed in Section 2, the relevant figure of merit that allows for the investigation of such quantity is ergotropy, as defined in Equation (13).

### 5.2. Quantum Many-Body Effects

As stated above, a qubit is the simplest example of QB. A natural question that arises is whether having a collection of interacting qubits can improve the performance of such devices due to *quantum collective effects*. Many studies in the literature have shown situations where this is indeed the case for both energy injection and extraction. For example, Alicki and Fannes [156] showed that, although each qubit in a collection of *N* identical qubits may individually be in a passive state, work can still be extracted from the composite system. This occurs because the unitary operations acting on the collection of qubits can be global, exploiting correlations among subsystems to better redistribute the populations across the many-body energy eigenstates, as discussed in Section 2. The extraction of useful work from an ensemble of subsystems proceeds until the system reaches a completely passive state from which no further work can be extracted by unitary operation.

Completely passive states provide a fundamental thermodynamic characterization of equilibrium, identifying configurations from which no work can be extracted, even when collective unitary operations are allowed. While this concept is essential to determine the maximum amount of energy that can be extracted in a QB, it does not address how efficiently such energy can be injected or manipulated in time. This motivates the introduction of the notion of quantum advantage, which in the context of QBs refers to the possibility of outperforming classical or single-cell strategies during the charging process by exploiting collective quantum effects. A natural benchmark to assess quantum advantage is the comparison between a collective charging protocol acting on an *N*-cell battery and a reference strategy in which each cell is charged independently and in parallel under the same local constraints. A genuine advantage is identified when collective operations lead to a superlinear scaling of the charging power with the system size. Importantly, such an advantage is a dynamical property and does not necessarily require the final battery state to be entangled, but rather relies on the ability to generate collective dynamics during the charging stage [37].

In QBs based on interacting spin systems, the collective behavior arises from intrinsic many-body interactions. Spin chains, in particular, provide a physically testable platform to explore whether quantum advantage persists when charging is mediated by local or finite-range interactions and how it depends on interaction range, dimensionality and the underlying many-body structure of the battery. One of the early works to investigate the potential applications of spin chains as QBs was conducted by Le et al. [38], who investigated spin-chain models with nearest-neighbor interactions and demonstrated how collective effects driven by intrinsic interactions can enhance charging performance compared to parallel protocols within the same control constraints. Beyond nearest-neighbor spin-chain models, cavity-mediated QBs have been proposed, in which long-range interactions induced by the cavity field enhance the charging performance and lead to superlinear scaling of the charging power, with exponents around or above 1.5, approaching quadratic behavior [168]. In particular, by studying a QB with the following Hamiltonian (particular case of the one in Equation (17))

(42) $$H_{B}=B\sum_{i=1}^{N}\sigma_{i}^{z}-\sum_{i<j}g_{ij}\left[\sigma_{i}^{z}⊗\sigma_{j}^{z}+\alpha \left(\sigma_{i}^{x}⊗\sigma_{j}^{x}+\sigma_{i}^{y}⊗\sigma_{j}^{y}\right)\right],$$

where *B* is the intensity of an external Zeeman field breaking degeneracy between spins, $g_{ij}$ represents the strength of the interaction between spins *i* and *j* (which can be short or long range), and α parametrizes the anisotropy. They found that, for sufficiently strong interactions, the power deposited into the QB increases beyond the extensive scaling expected from independent spins. This enhancement becomes especially pronounced in the presence of long-range interactions, where collective effects allow energy injected by the driving field to be redistributed efficiently across the entire chain. Importantly, the observed advantage arises from the interaction-induced collective dynamics during the charging process, rather than from entanglement in the final battery state. At the same time, the analysis clarified that the quantum advantage identified in this model is not universal, but depends sensitively on the interaction range and strength. For nearest-neighbor couplings, the charging power remains extensive in the system size, although interactions still affect the actual value of the power per spin and the charging time.

A complementary and conceptually distinct approach was introduced by Rossini et al. [169] in their work on Sachdev–Ye–Kitaev (SYK) QBs. In this model, the battery itself consists of *N* identical and noninteracting spin-1/2 cells, governed by a local Hamiltonian of the form

(43) $$H_{B}=\sum_{j=1}^{N}h_{j}^{\left(1\right)},$$

where $h_{j}^{\left(1\right)}$ denotes the single-site term introduced in Section 3.1, here specified as

(44) $$h_{j}^{\left(1\right)}=\omega_{0}\sigma_{j}^{y}.$$

Here, $\omega_{0}$ represents the local energy scale (or Zeeman splitting) of each spin. The ground state of $H_{B}$ corresponds to the fully discharged configuration of the battery. The charging process is implemented by suddenly switching off $H_{B}$ and activating, for a finite time interval τ, a global interaction Hamiltonian $H_{\mathrm{col}}$ according to

(45) $$H\left(t\right)=H_{B}+\lambda \left(t\right)\left(H_{\mathrm{col}}-H_{B}\right),$$

where λ(t) is a step function equal to unity for t∈[0,τ] and zero otherwise. In this specific model, the charging Hamiltonian acts on the same Hilbert space as the battery and does not represent an independent quantum charger; for this reason, we denote it by $H_{\mathrm{col}}$ to emphasize its role as a collective charging Hamiltonian. In particular, for this charging scheme, the Hamiltonian $H_{\mathrm{col}}$ is chosen to be the SYK model,

(46) $$H_{\mathrm{col}}^{\mathrm{SYK}}=\sum_{i,j,k,l=1}^{N}J_{ijkl}\;c_{i}^{†}c_{j}^{†}c_{k}c_{l},$$

where $c_{j}^{†}$ ($c_{j}$) are spinless fermionic creation (annihilation) operators and the couplings $J_{ijkl}$ are independent random variables with zero mean. Here, the spin degrees of freedom are mapped onto fermionic operators, as customary in SYK-based models. By exploiting the highly nonlocal and strongly interacting nature of the SYK charging Hamiltonian, the authors demonstrated a super-extensive (quadratic) scaling of the optimal charging power with *N*, thereby identifying a genuine quantum advantage. Importantly, this advantage originates from the highly entangling many-body dynamics generated during the charging stage, rather than from correlations present in the final battery state.

Extensions of the Dicke model, including direct interatomic interactions and external driving fields, further enrich the charging dynamics, leading to modified scaling of charging power and the emergence of critical behavior in the stored energy [170]. Across different interacting spin models, the physical origin of the quantum advantage exhibited by QBs can vary substantially, reflecting the richness of many-body mechanisms that can be exploited for energy storage and charging. In some cases, this enhancement is rooted in the structure of the charging dynamics itself. A notable example is provided by one-dimensional Kitaev-type spin models, where it has been shown that the ergotropy can display super-extensive scaling with the system size, signaling a genuine many-body quantum advantage tied to the interplay between anisotropy and interaction strength, which generates nontrivial correlations during the collective charging process [171]. In this setting, anisotropic couplings enable an efficient redistribution of energy across the chain that cannot be achieved by parallel charging protocols and does not rely on idealized global interactions.

More generally, the collective spin dynamics can be significantly modified by engineering additional coupling terms, offering alternative routes to enhanced battery performance. For instance, QBs based on Heisenberg spin chains supplemented by Dzyaloshinskii–Moriya interactions have been shown to exhibit increased ergotropy and charging power compared to their isotropic counterparts, demonstrating that spin-orbit-like couplings can act as an effective resource even in systems constrained by locality [172].

Beyond dynamical mechanisms, quantum advantage can also originate from the static spectral properties of the battery Hamiltonian. This perspective is exemplified by the concept of frustrated QBs, where competing interactions generate dense low-energy manifolds that facilitate work storage and extraction, highlighting a qualitatively distinct route to improved performance [41].

Related interaction-induced collective effects have also been identified in other multi-spin architectures. Studies of interacting two-level systems undergoing collective charging have shown that the nature of the interactions—repulsive or attractive—can markedly influence both the stored energy and the robustness against dissipation, reinforcing the central role played by interactions in shaping battery performance [173]. Moreover, even at the few-qubit level, optimized charging protocols for short spin chains have demonstrated that many-body structure and control design can lead to advantages over naive parallel strategies [174].

Recent studies have extended the investigation of integrable spin-chain QBs to the thermodynamic limit, focusing on how many-body phenomena such as quantum phase transitions influence energy storage and charging dynamics, revealing robust signatures of criticality in the charging process [42,43]. In particular, when the charging protocol crosses a quantum phase transition, the stored energy becomes largely insensitive to the precise charging parameters in the thermodynamic limit, highlighting a stable many-body effect that persists across a broad range of quench timescales. Moreover, nonanalytic features in the stored energy can appear at the critical points of the charging Hamiltonian’s phase diagram, even when the initial state is thermal.

Beyond quantum phase transitions, spin chains in the thermodynamic limit have been explored as QBs for additional reasons related to transport and dynamical properties. Disordered spin chains, in particular, provide a natural setting to investigate how Anderson and many-body localization affect energy storage and extraction. In the localized regime, the suppression of excitation spreading limits energy redistribution and reduces the achievable charging power even for large *N*, whereas ergodic phases allow for efficient transport and a larger conversion of injected energy into ergotropy [39,175]. These results emphasize that the performance of many-body QBs is not dictated solely by the total stored energy, but is strongly influenced by the underlying dynamical regime of the spin model. Along similar lines, in the presence of finite-time ramps and stochastic noise, transverse-field Ising chain QBs show a nontrivial interplay between interaction-induced correlations, noise and the charging protocol [176,177,178,179].

### 5.3. Open Quantum Batteries

In many practical implementations of spin-based QBs, interactions between the system and the environment cannot be ignored. Here, dissipation typically arises from phononic environments, electromagnetic modes or spin–spin coupling to uncontrolled degrees of freedom. This motivates the study of *open quantum batteries* (OQBs) [89,180], where the spin system employed as a QB is open, i.e., it can exchange energy (and possibly particles) with the environment. When OQBs are considered, the focus is not only on identifying efficient unitary charging and discharging protocols, but also on understanding how *dissipative effects* inherent to realistic spin platforms impact the performance of QBs. As discussed in Section 3.2, the full Hamiltonian includes, in addition to the battery $H_{B}$ and the charger $H_{C}$, an environment term $H_{E}$ and a battery–environment interaction term $H_{\mathrm{BE}}$.

The literature on OQBs has rapidly expanded in recent years, exploring both Markovian and non-Markovian regimes of system–environment interaction. In the Markovian regime, dissipation can itself be exploited as a resource to charge QBs, while also establishing fundamental bounds on charging power and efficiency [180,181,182]. In contrast, for non-Markovian OQBs, structured reservoirs or strong system–bath couplings give rise to dynamics that deviate from simple exponential relaxation, allowing partial recovery of coherence and energy during the charging process. Several studies have shown that non-Markovianity can enhance charging power, prolong ergotropy retention, and mitigate decoherence-induced losses when compared to purely Markovian dynamics [89,183,184,185]. From this perspective, spin systems provide a natural testbed to investigate how different dissipative regimes affect collective charging dynamics, thanks to their tunable interactions and well-established experimental control.

## 6. Experimental Implementations

The theoretical developments reviewed so far have been accompanied by a fast experimental progress, enabled by advances in quantum control, coherent manipulation and engineered dissipation in well-controlled spin platforms. Over the last decade, various physical platforms have emerged as leading candidates for the realization of spin-based QTMs and QBs, allowing direct tests of quantum thermodynamic principles at the single- and few-body level, as well as exploration of collective many-body effects.

In this section, we review the main experimental platforms where spin-based QTMs and QBs have been implemented or are actively being pursued, highlighting their respective strengths, limitations and relevance in view of the realization of scalable quantum technologies for energy manipulation.

### 6.1. Nuclear Magnetic Resonance Platforms

Nuclear magnetic resonance (NMR) platforms have played an important role in the experimental development of quantum thermodynamics by providing highly controllable spin systems operating at room temperature [186]. In NMR experiments, nuclear spins act as effective two-level systems whose coherent manipulation, initialization and readout can be achieved with exceptional precision using radio-frequency control techniques. The long coherence times and the high level of control available in NMR make these systems particularly well-suited for implementing and characterizing quantum thermodynamic protocols at the ensemble level.

While NMR was not the earliest experimental platform considered to test quantum thermodynamics, it has proven especially valuable for proof-of-principle demonstrations of information–energy conversion, quantum Maxwell demons and measurement-based thermodynamic protocols [187,188,189]. In these experiments, ensembles of nuclear spins were used to investigate the energetic cost of information processing, feedback control and entropy production, thereby establishing a direct operational connection between quantum thermodynamics and quantum information theory.

Beyond information-theoretic settings, NMR has also enabled direct experimental realizations of QTMs. In particular, spin-based quantum Otto engines have been implemented using NMR techniques, allowing full characterization of work and heat statistics, irreversibility and efficiency at finite time [190]. These experiments demonstrated how thermal and quantum fluctuations jointly contribute to entropy production and provided a controlled platform to study quantum engines operating at maximum power.

More recently, NMR has been employed to realize QTMs powered by measurements rather than conventional thermal reservoirs, where measurement back-action acts as an effective thermodynamic resource [191]. These experiments highlight the versatility of NMR platforms for exploring non-standard thermodynamic paradigms.

In parallel, NMR has emerged as a leading experimental platform for realizing small-scale QBs based on interacting spin architectures. In particular, star-topology NMR spin systems have enabled the first experimental investigations of QB charging, ergotropy extraction and energy consumption in a controlled setting [160]. These experiments demonstrated a quantum advantage in charging mediated by correlations between charger and battery spins and provided a concrete realization of charger–battery–load circuits at the nuclear-spin level.

### 6.2. Trapped Ions

Trapped ions constitute one of the most versatile and mature experimental realizations of spin-based quantum systems [192]. In these setups, long-lived internal electronic or hyperfine states of individual ions encode effective spin-1/2 degrees of freedom, while laser-driven interactions enable high-fidelity coherent control and tunable spin–spin couplings. Effective local or long-range interactions can be engineered through shared motional modes of the ion crystal, allowing precise implementation of both few-body and collective spin Hamiltonians under well-controlled conditions [193,194,195].

Quantum heat engines have been experimentally realized using single ions as working substances. In these experiments, controlled laser cooling and heating simulate thermal reservoirs, while unitary work strokes are implemented through coherent laser pulses acting on the internal states of the ion. Landmark demonstrations enabled direct measurement of work, heat and efficiency at the single-particle level, providing clear experimental validation of quantum thermodynamic concepts such as finite-time operation, coherence generation and irreversibility [196,197]. These experiments established trapped ions as a flagship platform for testing the foundations of quantum heat engines with unprecedented control and precision.

Beyond heat engines, trapped ions have also enabled pioneering experimental realizations of quantum refrigerators. A notable example is the demonstration of a quantum absorption refrigerator using three trapped ions, where three normal modes of motion are coupled by an effective trilinear Hamiltonian. In this autonomous device, heat exchange between two modes leads to refrigeration of a third mode without external work input. This experiment demonstrated cooling performance beyond classical benchmarks by exploiting quantum resources such as coherence and squeezing and provided access to both steady-state and single-shot refrigeration regimes [198]. This work highlighted the ability of trapped-ion-based devices to implement fully autonomous QTMs within a unified and controllable setting.

Trapped ions have recently emerged also as a promising platform for implementation of QBs. In particular, single-ion information engines have been considered in which the motional mode of the ion acts as the QB, while the internal spin degree of freedom controls cyclic charging and discharging through measurement and feedback. These experiments constitute the first fully cyclic realizations of information-driven QB charging in trapped-ion systems and explicitly link information thermodynamics to quantum energy storage [199]. Although current demonstrations focus on single-ion batteries, they represent a crucial experimental milestone for QBs in atomic platforms.

Trapped-ion chains also offer a natural and highly controllable route toward many-body QTMs and collective QBs. Long-range interactions mediated by collective motional modes allow theoretical and experimental exploration of interaction-enhanced power, collective charging protocols and non-equilibrium many-body effects in extended systems. At present, such collective QBs in ion chains remain largely at the proposal stage. Nevertheless, trapped ions remain one of the most promising platforms for realizing and testing many-body quantum thermodynamic advantages under controlled and scalable conditions [193,194,195].

### 6.3. Nitrogen-Vacancy Centers in Diamond

Nitrogen-vacancy (NV) centers in diamond provide a solid-state spin platform for quantum thermodynamics, combining long coherence times with optical initialization and readout at room temperature. The electronic ground state of the NV center forms an effective spin-1 system, which is often reduced to a two-level subspace for thermodynamic protocols, while nearby nuclear spins (such as ^14^N and ^13^C) and lattice phonons naturally act as structured environments. These features make NV centers particularly attractive for studying open-system dynamics and non-equilibrium thermodynamics in solid-state settings [200].

To date, the primary experimental realization of a QTM based on NV centers is the landmark demonstration of a microscopic quantum heat engine by Klatzow et al. [201]. In this experiment, an ensemble of negatively charged NV^−^ centers was driven through Otto-like thermodynamic cycles at room temperature using optical and microwave control. The study directly accessed work output, efficiency, entropy production and demonstrated how quantum coherence can enhance engine performance. This work established NV centers as a viable platform for implementing and characterizing quantum heat engines under non-equilibrium driving.

Beyond heat engines, NV centers have been extensively used to investigate foundational aspects of quantum thermodynamics related to measurement back-action, dissipation and information processing [202]. While full experimental realizations of quantum refrigerators or autonomous thermal machines based on NV centers are still lacking, theoretical and experimental studies have explored how controlled dissipation, spin–phonon coupling and measurement-induced dynamics can be harnessed as thermodynamic resources in these systems [200].

At present, experimental implementations of QBs using NV centers remain at the proposal stage. Nevertheless, the intrinsic robustness of NV spins, their compatibility with nanoscale environments and the availability of optical and microwave control make them promising candidates for future realizations of open QBs and autonomous QTMs in solid-state architectures. In this respect, NV centers complement platforms such as trapped ions and nuclear magnetic resonance, which currently host more advanced experimental demonstrations, by offering a scalable and room-temperature solid-state testbed for quantum energy technologies.

### 6.4. Superconducting Circuits

Superconducting circuits constitute a leading solid-state platform for quantum technologies, combining high-level controllability, fast dynamics and scalable architectures [203,204,205,206,207,208,209,210,211,212,213,214]. In these systems, electrical circuits playing the role of artificial atoms behave as effective two-level (spin-1/2) systems coupled to engineered electromagnetic environments within the framework of circuit quantum electrodynamics [215]. The high degree of tunability in system parameters and dissipation channels makes superconducting circuits particularly well suited for exploring quantum thermodynamic processes beyond idealized weak-coupling and Markovian regimes [216,217].

Superconducting platforms have enabled seminal experimental investigations of information thermodynamics and work extraction based on measurement and feedback. A prominent example in this direction is the experimental realization of a quantum Maxwell demon using a superconducting qubit, where information acquired through measurement was converted into extractable work, directly demonstrating information–energy conversion at the quantum level [218]. While such experiments do not constitute full cyclic heat engines, they provide direct access to work statistics, entropy production and feedback-controlled thermodynamic processes.

Experimental realizations of fully cyclic quantum heat engines remain comparatively more recent than in trapped-ion or NMR platforms. Nevertheless, dissipation-engineered superconducting devices have recently enabled proof-of-concept experimental demonstrations of cyclic quantum heat engine operation, where controllable reservoirs and engineered couplings allow reversible switching between refrigeration and work-producing modes [219]. These advances mark an important step toward QTM operating in solid-state architectures. Very recent experiments have also demonstrated autonomous quantum heat engines in superconducting circuits, where heat currents between engineered reservoirs generate coherent microwave radiation driven solely by thermal gradients [220].

In contrast, quantum-circuit refrigerators based on tunable superconducting tunnel junctions have achieved controlled cooling of microwave resonators and qubits, enabling autonomous refrigeration at the single-quantum level [221]. More recent experiments have demonstrated thermally driven quantum absorption refrigerators capable of autonomously resetting superconducting qubits to effective temperatures below those achievable with any single thermal bath, highlighting the practical relevance of such devices for quantum information processing [222].

Recent experiments have demonstrated superconducting QBs using multilevel circuit architectures. In particular, Hu et al. experimentally demonstrated a superconducting qutrit QB, directly measuring energy storage, ergotropy and self-discharge effects, thereby establishing a solid-state platform for quantum energy storage [162]. The same physics has also been investigated via accessible IBM quantum platforms [159]. Subsequently, Ge et al. realized efficient charging and discharging of a superconducting transmon-based QB using frequency-modulated STIRAP protocols, experimentally observing enhanced population transfer, ergotropy, and charging power [223]. Very recently, a scalable superconducting QB composed of up to 12 transmon qubits has been experimentally realized, demonstrating a clear quantum charging advantage and providing direct evidence of collective enhancement in solid-state architectures [224].

In parallel, several theoretical works have investigated the performance and optimization of superconducting QBs based on coupled qubit architectures. For instance, Elghaayda et al. proposed a superconducting QB model composed of interacting qubits and analyzed the role of Josephson energies, coupling strengths and coherence in optimizing ergotropy and power, providing experimentally feasible parameter regimes and design guidelines for future implementations [225].

Another important direction in superconducting platforms concerns QBs based on circuit QED architectures, where superconducting qubits or qutrits interact with microwave resonators [20,94,180,226]. In these systems, the photons trapped in the resonator can act as a quantum charger or as a mediator of effective interactions between artificial atoms. The resulting cavity-mediated coupling enables collective energy transfer and cooperative charging dynamics analogous to those described by the Dicke or Tavis–Cummings Hamiltonians [20]. Concrete implementations of such architectures include superconducting transmon qubit–resonator QBs, where dissipation channels and nearest-neighbor interactions can be engineered to stabilize energy storage and optimize charging power [227]. These architectures realize charger–battery schemes in which the cavity field injects energy into a spin ensemble and where the power is enhanced through cooperative effects. More recently, resonator–qutrit QBs have been investigated, showing that coherence and entanglement play distinct roles in charging, stability and self-discharge processes, with environmental noise potentially assisting stable energy storage under suitable conditions [228]. Related collective light–matter charging mechanisms have also been explored experimentally in cavity light–matter systems [22]. Due to the high degree of tunability of these platforms, including controllable coupling strengths and engineered dissipation channels, superconducting resonator–qubit systems represent a promising experimental testbed for studying collective QB charging and cavity-mediated quantum energy storage.

Overall, these experimental platforms investigate spin-based QTMs and QBs across complementary physical regimes. Nuclear magnetic resonance enables room-temperature ensemble control of effective spin systems; trapped ions provide high-fidelity manipulation of individual spin degrees of freedom; nitrogen-vacancy centers offer a solid-state platform for open-system spin thermodynamics; and superconducting circuits combine fast coherent control with scalable artificial-spin architectures. Together, these implementations demonstrate that spin-based QTMs and QBs have moved beyond purely theoretical proposals and now constitute experimentally accessible frameworks for quantum energy conversion and storage.

## 7. Conclusions

In this review, we have presented a comprehensive and unified perspective on *spin-based quantum energy devices*, encompassing both QTMs and QBs within the framework of quantum thermodynamics. By focusing on spin systems as controllable and experimentally accessible working media, we have highlighted how the same physical ingredients, such as discrete spectra, bounded energy levels, coherence, correlations and engineered dissipation, naturally enable both energy conversion and energy storage at the quantum level.

We began by revisiting the foundations of classical and quantum thermodynamics, emphasizing the operational definitions of heat, work, entropy and ergotropy that are essential for describing microscopic energy devices. Within this framework, spin systems emerge as particularly well-suited platforms due to their finite-dimensional Hilbert spaces, tunable Hamiltonians and compatibility with both unitary and dissipative control. These features enable a transparent connection between thermodynamic laws and microscopic dynamics, while also revealing the constraints imposed by passivity, bounded spectra, and quantum speed limits.

Building on these foundations, we reviewed spin-based QTMs, highlighting how cyclic, autonomous, and measurement-based architectures can be implemented using interacting spins. We showed that many-body interactions, quantum coherence and critical phenomena can qualitatively enhance machine performance, leading to increased power output, modified efficiency–power trade-offs, and novel operational regimes inaccessible to classical machines. In particular, collective effects and non-Markovian dynamics emerge as genuine thermodynamic resources, enabling transient energy backflow, coherence-assisted work extraction and stabilization of non-passive states. Measurement-based engines further illustrate how information acquisition and back-action can act as active thermodynamic ingredients, directly linking quantum measurement theory with energy conversion.

We then considered spin-based QBs, which provide the complementary functionality of energy storage and extraction. By characterizing these devices in terms of ergotropy rather than stored energy alone, we clarified the distinction between energetic excitation and extractable work, highlighting the central role of passivity. Spin chains and interacting spin models were shown to offer rich opportunities for collective charging protocols, where intrinsic interactions generate many-body dynamics capable of enhancing charging power beyond parallel single-cell strategies. Importantly, these quantum advantages are dynamical in nature and do not necessarily rely on entanglement in the final battery state, underscoring the relevance of interaction-driven collective evolution. Extensions to open QBs further revealed that dissipation and environmental memory effects, often viewed as detrimental, can instead be harnessed to stabilize stored energy or even assist charging under suitable conditions.

A key message emerging from this review is that QTMs and QBs should not be regarded as disjoint concepts. Rather, spin systems provide a natural bridge between energy conversion and energy storage, enabling integrated architectures in which work extracted from thermal gradients can be directly stored as ergotropy in a QB. Autonomous spin-based machines, where engines and batteries are coupled without external time-dependent control, exemplify this unification and point toward fully quantum-coherent energy-processing devices. This unified perspective suggests that future quantum technologies may not treat engines and batteries as separate components, but as dynamically interconnected elements within coherent spin-based energy architectures.

On the experimental side, we surveyed the rapid progress across leading platforms to investigate real or effective spin systems, including nuclear magnetic resonance, trapped ions, nitrogen-vacancy centers in diamond and superconducting circuits. Each platform offers distinct advantages in terms of control, coherence, scalability and operating regimes. While trapped ions and NMR systems currently host the most mature demonstrations of QTMs, superconducting circuits excel in autonomous refrigeration and QB implementations, and NV centers provide a robust solid-state testbed for open-system thermodynamics. Together, these platforms demonstrate that spin-based quantum energy devices are transitioning from theoretical proposals to experimentally accessible systems.

Despite this progress, several open challenges remain. From a theoretical perspective standpoint, a comprehensive understanding of the interplay between coherence, correlations, control costs and dissipation, especially in the many-body and non-Markovian regimes, is still developing. Establishing universal bounds that incorporate realistic control constraints and finite-time operation remains an important open problem. From an experimental standpoint, scaling QBs, integrating them with engines into unified architectures, and achieving robust operation under realistic noise and disorder remain key challenges. Addressing these challenges will require close interplay between theory, experiment and quantum control.

Overall, spin-based quantum energy devices offer a promising route toward scalable and controllable quantum technologies for energy manipulation. Beyond their potential technological relevance, they provide a powerful platform for probing foundational questions at the intersection of thermodynamics, quantum information and many-body physics. As experimental capabilities continue to advance, spin systems are poised to play a central role in shaping the emerging field of quantum energy.

## Data Availability

No new data were created or analyzed in this study.
